# Analysis of influencing factors of congenital heart disease in children in Luzhou, China: a case–control study

**DOI:** 10.3389/fmed.2026.1745800

**Published:** 2026-03-10

**Authors:** Changling Zhang, Yansha Pan, Min Yang

**Affiliations:** 1 Department of Pediatrics, Affiliated Hospital of Southwest Medical University, Luzhou, Sichuan, China; 2 Sichuan Provincial Clinical Medical Center for Birth Defects, Luzhou, Sichuan, China

**Keywords:** children, congenital heart disease, influencing factors, risk factor, viral infection

## Abstract

The current research aimed to analyze the influencing factors for congenital heart disease (CHD) in children based on a case–control study. From January 2020 to January 2022, 60 children with CHD under 6 years of age admitted to the Affiliated Hospital of Southwest Medical University were selected as the research group. Another 60 children matched for age and sex who underwent physical examinations in our hospital without CHD were selected as the control group. The disease classification of children with CHD was determined, and the related influencing factors were analyzed. Among 60 children with CHD, atrial septal defect was the highest among the single heart defects, accounting for 36.67%, followed by ventricular septal defect (25.00%), patent ductus arteriosus (11.67%), pulmonary stenosis (6.67%), tetralogy of Fallot (5.00%), and aortic stenosis (1.67%). The rates of two or three combined heart defects were 10.00 and 3.33%, respectively. A family history of CHD, a history of viral infection in early pregnancy, and a history of medication in early pregnancy were identified as independent risk factors for CHD.

## Introduction

Congenital heart disease (CHD) refers to structural or functional abnormalities of the heart and blood vessels that are present at birth (1). Ventricular septal defect (VSD), atrial septal defect (ASD), patent ductus arteriosus (PDA), and tetralogy of Fallot (TOF) are among the most common kinds of CHD (2). The primary effects of CHD in children include heart dysfunction, body cyanosis, and growth dysplasia (3). CHD is a major public health concern due to its complex and diverse forms and potentially serious consequences, which may lead to abortion, stillbirth, and neonatal death, or postnatal disability (4).

According tothe Global Burden of Disease (GBD) 2021 database, the global prevalence of CHD among children under 5 years of age exceeded 4.18 million cases (5, 6). A population-based cross-sectional study conducted in eastern China reported a total birth prevalence of CHD of 5.79 per 1,000 live births (7). Children with CHD are prone to serious complications, including heart failure, endocarditis, and lung infections, which may significantly impair their physical and mental health as well as their growth and development (8).

Recently, significant advancements have been achieved in the diagnosis and management of CHD (9). Nevertheless, given the substantial disease burden associated with CHD, researchers both domestically and internationally have progressively redirected their research endeavors toward exploring its etiology (10). Currently, a majority of studies posit that CHD arises from an interplay between genetic and environmental factors (11, 12). Genetic factors play a pivotal role in the pathogenesis of CHD and include single-gene mutations, polygenic disorders, chromosomal aberrations, and translocations (13). Environmental factors primarily involve maternal intrauterine infections, smoking and alcohol consumption during pregnancy, prenatal exposure to specific substances, prenatal medication use, and pregnancy-related illnesses (14). Research has demonstrated that maternal rubella infection, febrile illnesses, or influenza during gestation, as well as the use of certain medications in early pregnancy (such as retinoic acid, antiepileptic and anticonvulsant drugs, opioid analgesics, oral anticoagulants, and beta-blockers), are linked to an increased risk of CHD in offspring (15). Due to the differences in genes, lifestyle, and living environment of different regions, nationalities, and people, the risk factors of exposure are also different, and with the constant changes in living environment and lifestyle, the primary factors affecting the occurrence of CHD may also change (16). Therefore, understanding the risk factors for childhood CHD is particularly important for effective prevention and control of the disease in our province.

In this study, we analyzed the influencing factors of CHD in children based on a case–control study.

## Materials and methods

### Materials

A total of 60 children with CHD under 6 years of age admitted to the Affiliated Hospital of Southwest Medical University from January 2020 to January 2022 were included in the research group. The inclusion criteria for the research group were as follows: (1) children with a confirmed diagnosis of CHD; (2) children aged 3–6 years; (3) children without severe acute or chronic infectious diseases, genetic metabolic, or chronic wasting diseases; and (4) children whose guardians provided verbal consent for participation in the study and signed the informed consent.

The control group comprised 60 children without CHD who underwent physical examination at our hospital during the same period. Control group participants were selected through frequency matching based on age and sex to the research group cases. The inclusion criteria for the control group were as follows: (1) children with no CHD; (2) children aged 3–6 years; (3) children without cardiovascular disease, severe acute and chronic infectious diseases, and genetic metabolic and chronic wasting diseases; and (4) children whose guardian provided verbal consent for participation in the study and signed the informed consent.

This study was approved by the Ethics Committee of the Affiliated Hospital of Southwest Medical University.

### Survey methods

The three-level survey method of general physical examination, return visit, and diagnosis was adopted for the survey subjects. The guardians of all survey subjects were distributed the CHD Epidemiological Survey Questionnaire for preschool children, and trained personnel guided them to fill out the questionnaire. During physical examination, attention was focused on heart auscultation for murmurs or P2 hyperactivity, as well as percutaneous oxygen saturation (24 h monitoring of oxygen saturation in the right upper limb and either lower extremity; a reading of ≥95% in the right hand or any foot, with a difference between the two ≤3%), and children with one of the following conditions were taken as subjects for return visit: (1) The child’s anthropometric measurements (height and weight) fell below the 5th percentile, or physical fitness appeared significantly compromised, as assessed against the age- and sex-specific growth standards published by the World Health Organization (WHO) for children under 5 years and the Chinese national growth references for children aged 3–6 years; (2) a history of multiple upper respiratory tract infections; (3) auscultation of heart murmurs above grade II by attending physicians with more than 5 years’ working experience in this specialty; and (4) cyanosis of the nail bed and lip in the resting state. Echocardiography was performed to confirm the diagnosis.

### Survey contents

1) Demographic data included name, age, sex, ethnicity, home address, gestational age, and family history of CHD within fourth-degree relatives;
2) The participants were asked about their parents’ age, occupation, place of residence during pregnancy, whether they had been inbred, and genetic history of hypertension, smoking, and drinking;
3) The age of maternal pregnancy, the number of fetal pregnancies, the exposure to toxic substances (such as phenols, formaldehyde, and paint) in early pregnancy (the first 3 months of pregnancy), the history of viral infection in early pregnancy, and the history of medication use (such as thalidomide, vitamin A and derivatives, antiepileptic drugs, and non-steroidal anti-inflammatory drugs) in early pregnancy;
4) Nutritional characteristics of children, including height, weight, and body mass index (BMI); and.
5) The results of heart auscultation (presence of murmurs or P2 hyperactivity), percutaneous oxygen saturation (SpO₂), and the SpO₂ difference between the limbs.

### Statistical analysis

SPSS 21.0 was implemented to analyze the data, and the measurement data were expressed as mean ± standard deviation and compared by *t*-test. The statistical data were expressed as cases (*n*) or percentages (%), and the χ^2^ test was used for comparison. A logistic regression analysis was used to analyze the related influencing factors of CHD. A *p*-value of <0.05 was considered statistically significant.

## Results

### General clinical data analysis of children in two groups

The detailed comparisons between the research group and the control group are presented in Table 1. No significant differences were observed in sex, nationality, parental genetic history of hypertension, number of births, gestational age, or nutritional characteristics (height, weight, and BMI) between the two groups (*p* > 0.05). Statistically significant differences were observed in the family history of CHD, maternal exposure to second-hand smoke, exposure to toxic substances in early pregnancy, a history of viral infection in early pregnancy, a history of medication use in early pregnancy, and maternal age at pregnancy (*p* < 0.05). As expected, significant differences were also found in physical examination findings related to CHD. Children in the research group had a significantly higher prevalence of heart murmurs (grade ≥II) (80.00% vs. 3.33%, *p* < 0.001) and P2 hyperactivity (30.00% vs. 0%, *p* < 0.001) on auscultation. Their mean percutaneous oxygen saturation (SpO₂) was significantly lower (95.22% vs. 98.52%, *p* < 0.001), and the SpO₂ difference between the right upper limb and lower extremity was significantly greater (2.85% vs. 1.02%, *p* < 0.001).

**Table 1:** General clinical data analysis of children in two groups.

General clinical data	Control group (*n* = 60)	Research group (*n* = 60)	χ^2^/t	*P*
Sex			0.03	>0.05
Male	33 (55.00)	32 (53.33)		
Female	27 (45.00)	28 (46.67)		
Nation			0.34	>0.05
Han	58 (96.67)	59 (98.33)		
Minority	2 (3.33)	1 (1.67)		
Family history of CHD			5.93	0.01
Yes	2 (3.33)	10 (16.67)		
No	58 (96.67)	50 (83.33)		
Parental genetic history of hypertension			0.20	>0.05
Yes	12 (20.00)	14 (23.33)		
No	48 (80.00)	46 (76.67)		
Maternal exposure to second-hand smoke during pregnancy			5.06	0.02
Yes	11 (18.33)	22 (36.67)		
No	49 (81.67)	38 (63.33)		
Maternal age at conception (years)	26.37 ± 2.68	29.38 ± 3.06	5.73	<0.001
Maternal exposure to toxic substances in early pregnancy			4.62	0.03
Yes	4 (6.67)	12 (20.00)		
No	56 (93.33)	48 (80.00)		
Maternal history of viral infection in early pregnancy			4.68	0.03
Yes	6 (10.00)	15 (25.00)		
No	54 (90.00)	45 (75.00)		
Maternal history of medication in early pregnancy			6.11	0.01
Yes	7 (11.67)	18 (30.00)		
No	53 (88.33)	42 (70.00)		
Number of fetuses			0.22	>0.05
Singleton	48 (80.00)	50 (83.33)		
Multiple	12 (20.00)	10 (16.67)		
Gestational age			0.06	>0.05
Term (37–42 weeks)	50 (83.33)	51 (85.00)		
Preterm or post-term	10 (16.67)	9 (15.00)		
Height (cm)	102.52 ± 8.13	100.83 ± 7.92	1.13	>0.05
Weight (kg)	16.82 ± 2.52	16.24 ± 2.31	1.38	>0.05
Body mass index (kg/m^2^)	16.02 ± 1.42	15.96 ± 1.58	0.38	>0.05
Heart murmur on auscultation			68.57	<0.001
Present (grade ≥II)	2 (3.33)	48 (80.00)		
Absent	58 (96.67)	12 (20.00)		
P2 hyperactivity on auscultation			42.19	<0.001
Present	0 (0.00)	18 (30.00)		
Absent	60 (100.00)	42 (70.00)		
Percutaneous oxygen saturation (SpO₂)	98.52 ± 1.04	95.22 ± 3.87	6.49	<0.001
SpO₂ difference between the right upper limb and the lower extremity	1.02 ± 0.52	2.85 ± 1.62	8.67	<0.001

### CHD-type composition ratio

As shown in Table 2, among 60 children with CHD, ASD was the highest in the single heart defect, accounting for 36.67%, followed by VSD (25.00%), PDA (11.67%), pulmonary stenosis (PS, 6.67%), TOF (5.00%), and aortic stenosis (AS, 1.67%), respectively. The rates of combined two or three heart defects were 10.00 and 3.33%, respectively.

**Table 2:** CHD-type composition ratio.

Disease type	Male (*n* = 32)	Female (*n* = 28)	Total
ASD	10	12	22 (36.67)
VSD	9	6	15 (25.00)
PDA	4	3	7 (11.67)
PS	2	2	4 (6.67)
TOF	2	1	3 (5.00)
AS	1	0	1 (1.67)
Combined with two heart defects	3	3	6 (10.00)
Combined with three heart defects	1	1	2 (3.33)
Total	32	28	60 (100.00)

### Analysis of influencing factors of CHD

CHD was used as the dependent variable, and the mother’s age at pregnancy, CHD family history, pregnant woman’s exposure to second-hand smoke, toxic substance exposure in early pregnancy, a history of virus infection in early pregnancy, and a history of medication use in early pregnancy were considered as independent variables. A univariate logistic regression analysis was conducted, and it was found that family history of CHD, history of exposure to second-hand smoke in pregnant women, exposure to toxic substances in early pregnancy, a history of viral infection in early pregnancy, and a history of medication use in early pregnancy were potential risk factors for CHD (Table 3). Subsequently, the five suspected risk factors were screened by the multivariate logistic regression analysis. Family history of CHD, a history of viral infection in early pregnancy, and a history of medication use in early pregnancy were independent risk factors for CHD (*p* < 0.05), as shown in Table 4.

**Table 3:** Single logistic regression analysis of CHD influencing factors.

Risk factors	*β*	Standard error	Wald χ^2^	OR (95% CI)	*P*
Family history of CHD	2.23	0.13	7.86	2.12 (1.33–4.03)	<0.001
Maternal exposure to second-hand smoke during pregnancy	1.49	0.66	6.05	1.84 (1.06–2.92)	0.01
Maternal exposure to toxic substances in early pregnancy	3.02	1.23	10.68	2.31 (1.53–4.22)	<0.001
History of viral infection in early pregnancy	1.46	0.62	5.87	2.82 (1.95–3.73)	<0.001
History of medication use in early pregnancy	2.53	1.05	9.16	2.33 (1.43–4.25)	0.02

**Table 4:** Multivariate logistic regression analysis of CHD influencing factors.

Risk factors	*β*	Standard error	Wald χ^2^	OR (95% CI)	*P*
Family history of CHD	3.25	1.74	9.76	4.92 (2.33–9.89)	<0.001
History of viral infection in early pregnancy	2.71	1.75	11.86	4.15 (2.24–8.32)	<0.001
History of medication use in early pregnancy	1.42	0.51	8.26	3.08 (2.43–4.35)	<0.001

## Discussion

CHD is the most common and serious congenital malformation in the clinic, accounting for approximately one-third of congenital malformations (17). In our study, among 60 children with CHD, ASD was the highest in the single heart defect, accounting for 36.67%, followed by VSD (25.00%), PDA (11.67%), PS (6.67%), TOF (5.00%), and AS (1.67%), respectively. The rates of combined two or three heart defects were 10.00 and 3.33%, respectively. At present, surveys and studies have shown that ASD, VSD, and PDA are among the top three CHD types (18), which is in accordance with our research findings.

Given the serious harm caused by CHD, identifying its etiology is positively significant for guiding clinical intervention measures as soon as possible and for preventing the occurrence of CHD. It is widely believed that genetics, environment, and gene–environment interaction are the three primary causes of CHD (19). Among these causes, the gene–environment interaction factor is the most important cause of CHD (20). In recent years, numerous clinical studies exist on the etiology of CHD, which can be summarized into three primary causes, namely maternal factors, genetic factors, and physical and chemical factors, such as infectious pathogens that can cause vertical transmission between mother and child, irrational drug use during pregnancy, single gene/multiple gene mutations, smoking, and chemical exposure (21). In our study, using the multivariate logistic regression analysis, we found that family history of CHD, a history of viral infection in early pregnancy, and a history of medication use in early pregnancy were independent risk factors for CHD. It has become a medical consensus that CHD is related to genetic factors (22). Chromosome karyotype abnormality, chromosome copy number variation, single gene mutation, and polygene genetic defect are all associated with CHD (23). The more genes a CHD patient has, the more severe the disease. Family members who have a close relationship with the patient are more likely to suffer from this disease than those with a distant relationship (24). Families with more genes possess a higher risk of developing CHD and have a larger number of patients (25). Besides, studies have shown that a history of respiratory virus infection—such as rubella virus, Coxsackie virus, herpes simplex virus, and cytomegalovirus—in early pregnancy can lead to cardiovascular dysplasia, in which rubella virus is the leading cause of CHD (26). It has been reported that the occurrence of CHD is associated with influenza virus infection (27). Additionally, studies have shown that sulfonamides in antibiotics can significantly affect the normal cardiovascular development of embryos (28). In addition, antidepressants, anti-asthmatics, antifungal medications, medications for hypothyroidism, and hypertension can increase the risk of CHD (29). All of the abovementioned literature support the findings of our study.

## Conclusion

Family history of CHD, a history of viral infection in early pregnancy, and a history of medication use in early pregnancy are independent risk factors for CHD. Therefore, preventive measures should be taken to improve the quality of healthcare during pregnancy, and early detection and timely intervention should be carried out for children with CHD.

## Data Availability

The original contributions presented in the study are included in the article/supplementary material, further inquiries can be directed to the corresponding author.
